# Electrochemical Behavior of β-Cyclodextrin-Ni-MOF-74/Reduced Graphene Oxide Sensors for the Ultrasensitive Detection of Rutin

**DOI:** 10.3390/molecules28124604

**Published:** 2023-06-07

**Authors:** Li Zhang, Mengting Zhang, Pingping Yang, Yin Zhang, Junjie Fei, Yixi Xie

**Affiliations:** 1 Hunan Engineering Laboratory for Preparation Technology of Polyvinyl Alcohol Fiber Material, College of Chemistry and Materials Engineering, Huaihua University, Huaihua 418000, China; zhanglicsu2013@163.com (L.Z.); yangpingping202211@163.com (P.Y.); 2 Key Laboratory of Research and Utilization of Ethnomedicinal Plant Resources of Hunan Province, Huaihua University, Huaihua 418008, China; 3 Hunan Provincial Higher Education Key Laboratory of Intensive Processing Research on Mountain Ecological Food, Huaihua 418008, China; 4 Key Laboratory of Environmentally Friendly Chemistry and Applications of Ministry of Education, College of Chemistry, Xiangtan University, Xiangtan 411105, China; 5 Key Laboratory for Green Organic Synthesis and Application of Hunan Province, Xiangtan University, Xiangtan 411105, China; 18371416942@163.com; 6 Junior Education Department, Changsha Normal University, Changsha 410100, China; zhangyincsu@163.com

**Keywords:** electroanalysis, carbon materials, electrochemical reduction, modified electrode

## Abstract

Rutin, as a biological flavonoid glycoside, has very important medicinal value. The accurate and rapid detection of rutin is of great significance. Herein, an ultrasensitive electrochemical rutin sensor based on β-cyclodextrin metal–organic framework/reduced graphene oxide (β-CD-Ni-MOF-74/rGO) was constructed. The obtained β-CD-Ni-MOF-74 was characterized by X-ray diffraction spectroscopy (XRD), X-ray photoelectron spectroscopy (XPS), scanning electron microscopy (SEM), Fourier transform infrared spectroscopy (FT-IR), and nitrogen adsorption and desorption. The β-CD-Ni-MOF-74/rGO presented good electrochemical properties benefiting from the large specific surface area and good adsorption enrichment effect of β-CD-Ni-MOF-74 and the good conductivity of rGO. Under optimal conditions for the detection of rutin, the β-CD-Ni-MOF-74/rGO/GCE showed a wider linear range (0.06–1.0 μM) and lower detection limit (LOD, 0.68 nM, (S/N = 3)). Furthermore, the sensor shows good accuracy and stability for the detection of rutin in actual samples.

## 1. Introduction

Rutin (3′,4′,5,7-tetrahydroxyflavone-3-d-rutin; RU), as a biological flavonoid glycoside, has very important medicinal value and plays an active role in anti-cancer, anti-diabetes, anti-ulcer, anti-hypertension, anti-aging, etc. An appropriate amount of RU has certain benefits for the human body. However, in the environment, rutin interacts with heavy metal ions to form harmful substances. In addition, excessive intake of rutin has adverse effects on human health [1,2,3]. Therefore, the sensitive detection of rutin is particularly important. At present, many technologies have been established and applied to detect rutin, including chemiluminescence (capillary electrophoresis electrochemiluminescence) [4], chromatography/mass spectrometry [5,6], and spectrophotometry [7]. However, these methods usually employ expensive instruments and have complex and time-consuming processes, which limit their applications in the areas of rapid and onsite sensing. In recent years, electrochemical sensors have attracted widespread attention due to their advantages such as simple operation, fast response, high sensitivity, and low cost [8,9,10]. For example, Li et al. [11] constructed a rutin electrochemical sensor based on Al-MOF/MWCNT-COOH, and Al-MOF/GCE had the lowest response value due to its low electron conduction efficiency. With the addition of carbon material, the composite material modified electrodes have greatly improved rutin-detection performance. Therefore, constructing a new type of electrochemical sensor to achieve the efficient and sensitive detection of RU is of great significance.

Metal–organic frameworks (MOFs) are a kind of porous coordination polymer composed of metal centers and organic ligands. Recently, Ni-MOF-74 has received widespread attention due to its high specific capacitance and excellent cycling stability [12]. For example, Shen et al. constructed a highly sensitive and selective sensor based on Pd-Ni-MOF-Ni, which achieved efficient chlorine removal due to the adsorption and catalytic performance of Ni-MOF [13]. However, the poor conductivity and stability of pure MOFs directly limit their application in the electrochemical field [14]. β-cyclodextrin (β-CD) is a cyclic oligosaccharide, which is composed of seven units of glucose. There are a large number of hydrophilic hydroxyl groups at the two cavity edges, which makes it easy to form hydrogen bonds between molecules to obtain self-assembly [15]. At the same time, inclusion complexes are formed with a large number of substrate molecules through host–guest non-covalent interactions [16]. Due to its advantages such as moderate cavity size, host–guest recognition, and low cost, it has been widely used in various fields [17,18]. It is worth mentioning that β-CD and its derivatives (β-CDs) are commonly used as molecular recognition and enrichment units in the field of electrochemical sensing [19,20,21,22]. For example, Zhao et al. constructed a quercetin electrochemical sensor by using rGO-CD nanoparticles [23]. Zhang et al. prepared a quercetin electrochemical sensor by using rGO-β-CD/GQDs/MoO_3_ composite [24]. Therefore, the composite materials formed by MOFs and CDs not only provide a hybrid nanomaterial but also have the potential to bring new properties, functions, and applications. However, β-CD/MOFs with poor conductivity limit their widespread application as electrochemical sensors. By improving the conductivity of the material and combining its advantages such as large specific area and good adsorption performance, the new composite material obtained is more suitable for constructing electrochemical sensors.

Graphite oxide (GO) is a typical two-dimensional carbon-based nanosheet. Due to its unique two-dimensional structure, good biocompatibility and large surface structures, such as planes, defects, and folds, it is an ideal carrier to improve the efficiency of electrocatalysis and electron transfer. In addition, due to its multiple oxygen-containing groups (-COOH, -CO, -OH, and epoxy groups), GO has good hydrophilicity [25,26,27,28]. After chemical or electrochemical reduction, reduced graphene oxide (rGO) is obtained [29,30]. Compared with GO, rGO has higher conductivity and a wider range of sensing applications [31,32,33,34,35,36]. Meanwhile, the residual oxygen-containing functional groups generated during the oxidation/reduction process can enhance the catalytic activity of rGO, and the defects in rGO can better transport electrons, which helps to improve electrochemical performance [37]. However, the direct reduction of GO on the electrode surface is considered a more effective and environmentally friendly method [38,39]. For example, Wang et al. [39] demonstrated that the direct reduction of GO on the electrode surface exhibited better detection performance for DA than the sensor using chemically reduced GO. 

Herein, β-CD-Ni-MOF-74 was synthesized by a hydrothermal method and compounded with GO by an electrochemical method to further synthesize β-CD-Ni-MOF-74/rGO composite materials. An ultrasensitive electrochemical rutin sensor based on β-CD-Ni-MOF-74/rGO was constructed. Due to the excellent enrichment ability of β-CD and the large specific surface area of Ni-MOF-74. In addition, the introduction of rGO increases the stability and conductivity of the composite material and the synergistic effects of these materials. The sensor exhibited good detection for rutin with good anti-interference ability, stability, and reproducibility, and was applied to actual samples.

## 2. Results and Discussion

### 2.1. Materials Characterization

**Figure 1:**
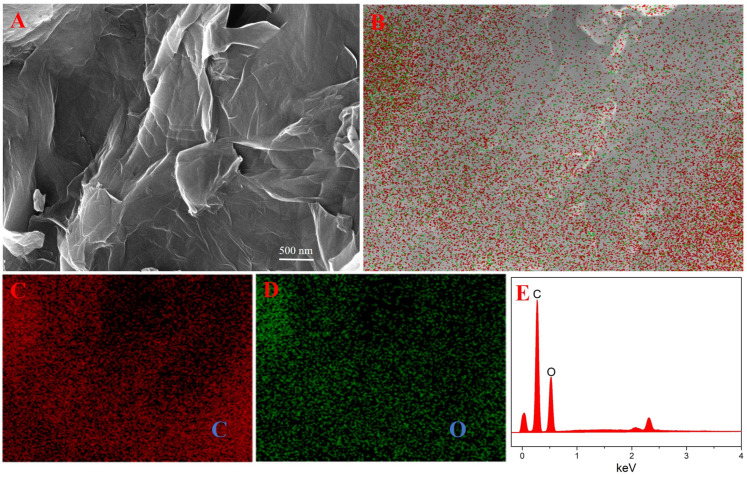
SEM images of (**A**) GO, (**B**–**E**) EDX of GO.

**Figure 2:**
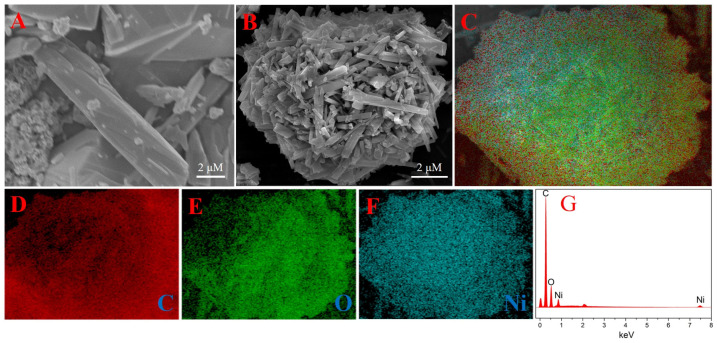
SEM images of (**A**) Ni-MOF-74, (**B**) β-CD-Ni-MOF-74, and (**C**–**G**) EDX of β-CD-Ni-MOF-74.

Scanning electron microscopy (SEM) was used to characterize the microstructure of Ni-MOF-74, GO, and β-CD-Ni-MOF-74. In Figure 1A, layered structures with obvious folds can be observed. In addition, the EDX images of GO, as shown in Figure 1B–E, indicate the successful synthesis of GO. Figure 2A,B show the very clear shapes and structures of the Ni-MOF-74 and β-CD-Ni-MOF-74 composites and Ni-MOF-74 exhibits a rod-shaped structure, which is consistent with the literature [40]. The rod-shaped structure of MOF-74 still remains after the β-CD is grown in situ on Ni-MOF-74, indicating that the doping of β-CD does not have a significant impact on the overall structure of Ni-MOF-74, and the resulting composite is still a rod-like structure with a small width. In addition, the EDX images of β-CD-Ni-MOF-74, as shown in Figure 2C–G, indicate the successful synthesis of β-CD-Ni-MOF-74.

**Figure 3:**
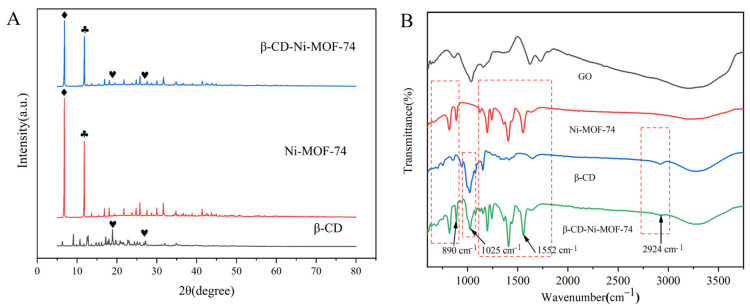
XRD patterns of β-CD, Ni-MOF-74, and β-CD-Ni-MOF-74 (**A**); the FT-TR spectra of GO, β-CD, Ni-MOF-74, and β-CD-Ni-MOF-74 (**B**).

The crystal plane of the material was characterized using X-ray diffraction (XRD) (Figure 3A). Compared with the simulation curve of Ni-MOF-74, the profiles of Ni-MOF-74 and β-CD-Ni-MOF-74 present good accordance [40]. The results indicate that Ni-MOF-74 with a good crystal structure was formed in both materials, which is consistent with the literature [41]. In addition, the characteristic diffraction peaks at 18.9° and 27.0° both belong to β-CD, which indicates that the introduction of β-CD does not change the crystal structure of Ni-MOF-74, and the composite material β-CD-Ni-MOF-74 was successfully synthesized.

Subsequently, Fourier transform infrared spectroscopy (FT-IR) was used to characterize the structure of different materials. As shown in Figure 3B, there are some absorption peaks of β-CD-Ni-MOF-74 appearing at 3000–3500 cm^−1^, 2924 cm^−1^, and 1025 cm^−1^, corresponding to hydroxyl (-OH), methylene (-NH_2_), and ether bonds (-C=O), respectively, and the above peaks are consistent with the characteristic peaks of β-CD. In addition, the peaks at 1552 cm^−1^ and 890 cm^−1^ correspond to the benzene ring oscillation peak and C-H oscillation peak, respectively [42], and those peaks are consistent with the characteristic peaks of Ni-MOF-74. All the results indicate that β-CD-Ni-MOF-74 was successfully synthesized.

**Figure 4:**
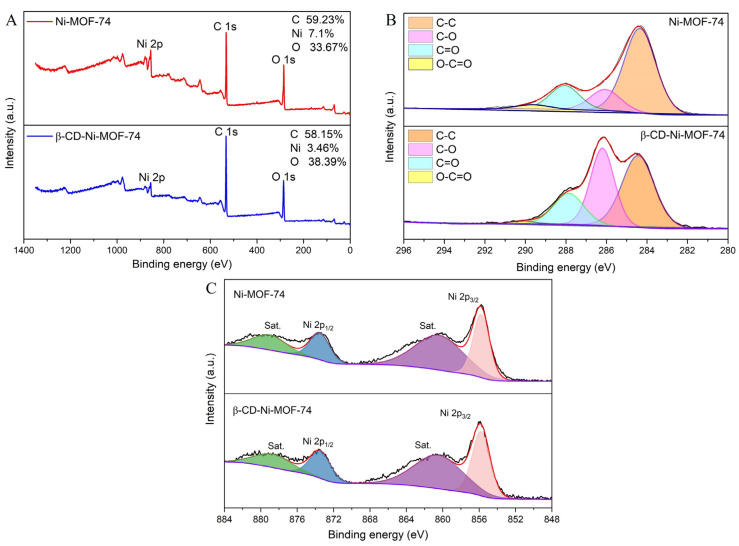
The XPS spectra (**A**) the survey spectrum; (**B**) C 1s spectrum; (**C**) Ni 2p spectrum.

The elemental compositions of the Ni-MOF-74 and β-CD-Ni-MOF-74 were characterized by X-ray photoelectron spectroscopy (XPS). The elemental compositions of the Ni-MOF-74 and β-CD-Ni-MOF-74 were characterized by XPS. The curves of Ni-MOF-74 and β-CD-Ni-MOF-74 (Figure 4A) both show that they consist of Ni, C, and O elements. As is shown in Figure 4B, the high-resolution XPS spectrum of C 1s shows several peaks at 284.7, 286.1, 288.1 eV, and 289.9 eV, belonging to C-C bonds, C-O bonds, C=O, and O-C=O, respectively. In addition, it can be clearly seen that the content of C-O in β-CD-Ni-MOF-74 is greatly increased, which may be due to the formation of more C-O bonds due to the in situ growth of β-CD and Ni-MOF-74. In the Ni 2p XPS spectrum (Figure 4C), two peaks can be seen at 856.4 and 873.2 eV, which belong to Ni 2p3/2 and Ni 2p1/2, respectively [43]. The peaks at 860.4 eV and 880.3 eV belong to the Sat.

**Figure 5:**
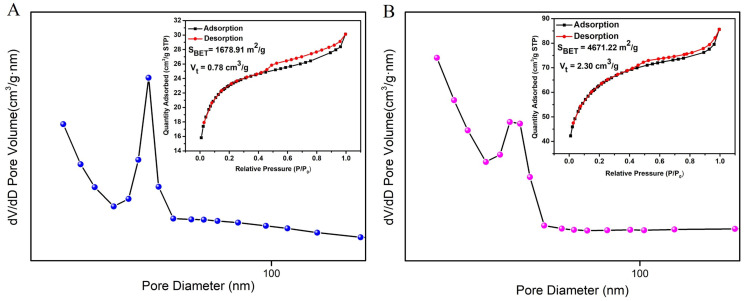
N_2_ adsorption−desorption isotherms (inset) and pore size distribution of (**A**) Ni-MOF-74 and (**B**) β-CD-Ni-MOF-74.

Subsequently, the pore distribution and specific surface area of the synthesized materials were investigated by nitrogen adsorption/desorption curves, where the typical IV curves were found in the β-CD-Ni-MOF-74 and Ni-MOF-74 (Figure 5). In the range of 0 < P/P0 < 0.45, the curve first rises rapidly and then slowly. In the range of 0.45 < P/P0 < 1, the isotherm rises faster, accompanied by an adsorption hysteresis loop.

After the in situ growth of β-CD, the specific surface area increased from 1678.91 m^2^/g to 4671.22 m^2^/g and the pore volume increased from 0.78 m^3^/g to 2.30 m^3^/g, which indicates the formation of more mesoporous structures. In addition, β-CD-Ni-MOF-74, with a larger specific surface area and pore volume, is beneficial for mass transfer, abundant active site exposure, and accelerating contact with the reaction species.

### 2.2. Electrochemical Characterization of Different Modified Electrodes

**Figure 6:**
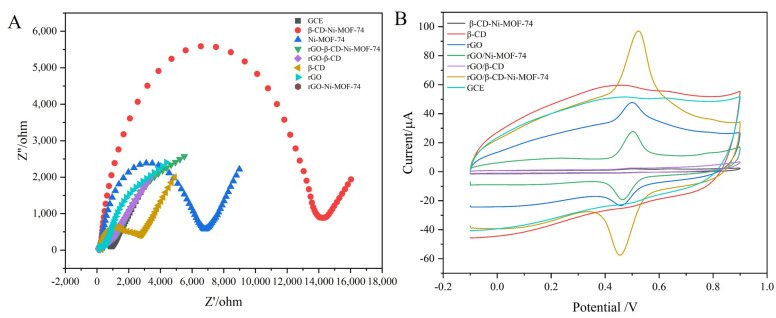
(**A**) EIS spectra of bare GCE, rGO/GCE, β-CD/GCE, rGO/β-CD/GCE, Ni-MOF-74/rGO/GCE, β-CD-Ni-MOF-74/GCE, and β-CD-Ni-MOF-74/rGO/GCE. Supporting electrolyte: 0.1 M KCl containing 5 mM Fe(CN)_6_^3−/4−^; frequency range: 0.1 Hz to 100 KHz. (**B**) CV of 2 μM RU on bare GCE, rGO/GCE, β-CD/GCE, rGO/β-CD/GCE, Ni-MOF-74/rGO/GCE, β-CD-Ni-MOF-74/GCE, and β-CD-Ni-MOF-74/rGO/GCE in 0.1 M PBS (pH = 3.5).

The conductivity of different modified electrodes was evaluated by electrochemical impedance spectroscopy (EIS). In [Fe(CN)_6_]^3−/4−^ solution, the Nyquist diagrams of different modified electrodes (bare GCE) are shown inFigure 6A, where the curve consists of a semicircle and a straight line. The smaller the semicircle, the smaller the charge transfer resistance (Rct) and the higher the conductivity. Figure 6A shows that β-CD/GCE and Ni-MOF-74/GCE both have a larger Rct, which indicates that β-CD/GCE and Ni-MOF-74/GCE have poor conductivity. With the introduction of β-CD, the Rct of the β-CD-Ni-MOF-74/GCE is the largest, which indicates that β-CD-Ni-MOF-74/GCE has the worst conductivity. However, β-CD, with good adsorption enrichment performance, improving the RU-detection performance. With the addition of rGO, the conductivity of rGO/β-CD-Ni-MOF-74, rGO/Ni-MOF-74, and rGO/β-CD was significantly improved. Good conductivity is more conducive to electron transmission and β-CD has good adsorption and enrichment performance. The rGO/β-CD-Ni-MOF-74/GCE exhibited high-performance detection of rutin.

Subsequently, cyclic voltammetry (CV) was used for RU detection on different modified electrodes. As shown in Figure 6B, β-CD-Ni-MOF-74/rGO/GCE obtained the maximum oxidation peak current (ipa), which is 2.7 times higher than Ni-MOF-74/rGO/GCE in a 0.1 M (pH = 3.5) PBS solution containing 2 µM RU. Compared to modified electrodes made of other materials, the β-CD-Ni-MOF-74/rGO/GCE obtained the largest ipa. The main reasons are as follows: on the one hand, β-CD has a good enrichment ability. After the surface of Ni-MOF-74 is coated with β-CD, its specific surface area increases, and it has more active sites, which improves the response of the composite to rutin. On the other hand, as the base material, β-CD-Ni-MOF-74 was fixed on the surface of GCE using the electrodeposition method, and GO was electrodeposited onto the surface of the material. However, the electrochemical reduction of GO can improve the electron transmission speed at the electrode interface compared with the modification of the traditional chemical reduction method. In addition, in an acidic environment, it provides more -COOH groups, which interact with the rich hydroxyl groups on the surface of β-CD to form strong hydrogen bonds and enhance the electron transmission capacity.

### 2.3. Optimization of β-CD-Ni-MOF-74 Concentration

The amount of β-CD-Ni-MOF-74 loaded by deposition affects the response of the modified electrode to RU. Therefore, we studied the influence of different number of electrodeposition on RU response. Different electrodeposition cycles (10, 15, 20, 25, 30, 35, and 40 cycles) were investigated to obtain the optimal detection ability of composite materials for RU. As shown in Figure S3A, as the number of electrodeposition cycles ranges from 10 to 25, the ipa gradually increases. When the number of electrodeposition cycles is 25, the sensor obtains the maximum ipa and achieves the best detection of RU. On the contrary, as the number of electrodeposition cycles continues to increase (25–40), the ipa gradually decreases. Due to the excessive content of β-CD-Ni-MOF-74, the film formed is thicker, which is not conducive to the transmission of electrons on the electrode surface. Therefore, we chose 25 cycles as the most optimal number of electrodeposition cycles.

### 2.4. Optimum Determination Conditions

Subsequently, the effects of different pH values (3.0, 3.5, 4.0, 4.5, 5.0, 5.5, 6.0, 6.5, 7.0, 7.5, 8.0) on the redox peak potential (Epa and Epc) of β-CD-Ni-MOF-74/rGO/GCE were studied by CV in 0.1 M PBS of 2 µM rutin. As shown in Figure S1, when the pH increases from 3.0 to 3.5, the ipa gradually increases. When pH = 3.5, the ipa is at the maximum value, indicating that the binding effect of β-CD-Ni-MOF-74/rGO and rutin is the strongest and achieving optimal detection performance. The response current gradually decreases when the pH changes from 3.5 to 8.0. So, pH = 3.5 is optimal (Figure S1C). In addition, when pH increases, the E_pa_ and E_pc_ both moved negatively, which indicates the involvement of protons in the electrochemical reaction. The pH exhibits a linear relationship with the E_pa_ and E_pc_ within a certain pH range (3.0–8.0) (Figure S1B). The linear equation is as follows:

E_pa_ = −0.0606 pH + 0.7138 (R^2^ = 0.9993)

E_pc_ = −0.0616 pH + 0.6881 (R^2^= 0.9977)

E^θ^ = −0.0611 pH + 0.9994 (R^2^= 0.9986)

The slope of the pH and E^θ^ is 61.1 mV·pH^−1^, which is close to the theoretical value of 59 mV·pH^−1^. According to the Nernst equation [44],

$$\frac{dEp}{dpH}=\frac{2.303mRT}{nF}$$

The ratio of protons to electrons (m:n) is about 1, which proves that the reaction of RU on the surface of β-CD-Ni-MOF-74/rGO/GCE is an “isoelectron–isoproton” process. The mechanism of RU on β-CD-Ni-MOF-74/rGO is shown in Scheme S1.

The adsorption capacity of the detection substance on the surface of the composite-modified electrode can be increased by enrichment potential and enrichment time, thereby improving the sensitivity of the sensor. The cavity of β-CD makes its enrichment ability more excellent. The enrichment potential and time of the composite were studied by differential pulse voltammetry (DPV). When the enrichment potential changes from −0.3 V to −0.2 V, the ipa shows an upward trend and reaches its maximum value at −0.2 V. When the enrichment potential changes from −0.2 V to 0.3 V, the ipa continuously decreases, so −0.2 V is the optimal enrichment potential (Figure S3B). Similarly, as the enrichment time increases from 1 min to 13 min, the ipa gradually increases and reaches its maximum value at 13 min. After 13 min, the ipa tends to flatten and slightly decrease, indicating that the adsorption capacity of the composite material reaches its maximum at 13 min (Figure S3C). Therefore, 13 min is the optimal enrichment time.

### 2.5. Electrochemical Behavior of β-CD-Ni-MOF-74/rGO/GCE at Different Scan Rates

The electrochemical behavior of RU on β-CD-Ni-MOF-74/rGO/GCE at different scan rates (V) was also studied by CV in 0.1 M PBS (2 μM RU) (Figure S2A). As the scanning rate increases from 20 V·s^−1^ to 400 V·s^−1^, the ipa and ipc also increase linearly, and the E_pa_ and E_pc_ move in the opposite direction. In addition, the ipa and ipc were linear with the sweep speed (V), but not proportional to the square root of the sweep speed (V^1/2^), which indicates that the electrode reaction of RU on β-CD-Ni-MOF-74/rGO/GCE was adsorption-controlled rather than diffusion-controlled. The relationships between the V and the peak current can be expressed by the following equations (Figure S2B):

ipa (μA) = −0.0757 ν (mV·s^−1^) − 0.6478 (R^2^ = 0.991)

ipc (μA) = 0.0893 ν (mV·s^−1^) + 1.8599 (R^2^ = 0.996)

The above results show that the potential changes with increasing V, demonstrating that the electron transfer of RU on β-CD-Ni-MOF-74/rGO/GCE is an irreversible redox process. The relationship between the V and E_pa_ can be expressed by the following equation (Figure S2C):

E_pa_ (V) = 0.0108 ln*v* (mV/s) + 0.4464 (R^2^ = 0.998)

According to the Laviron equation [45]:

$$Epa(V)=E^{\theta }+(\frac{RT}{\alpha nF})ln(\frac{RTk^{\theta }}{\alpha nF})+\left(\frac{RT}{\alpha nF}\right)lnv$$

where R, T, and F are constants that represent the universal gas constant, absolute temperature, and Faraday constant, respectively. In addition, α and n represent the transfer coefficient and electron number, respectively. Obviously, the n can be calculated according to the Laviron equation and E_pa_-lnv equation. According to the Laviron equation and E_pa_-lnv equation with a slope of 0.0108, n can be calculated (n = 2), which indicates that two electrons of RU are transferred during the electrochemical process on the surface of β-CD-Ni-MOF-74/rGO/GCE. This result is consistent with the literature [41].

### 2.6. Quantitative Analysis of RU on β-CD-Ni-MOF-74/rGO/GCE

**Figure 7:**
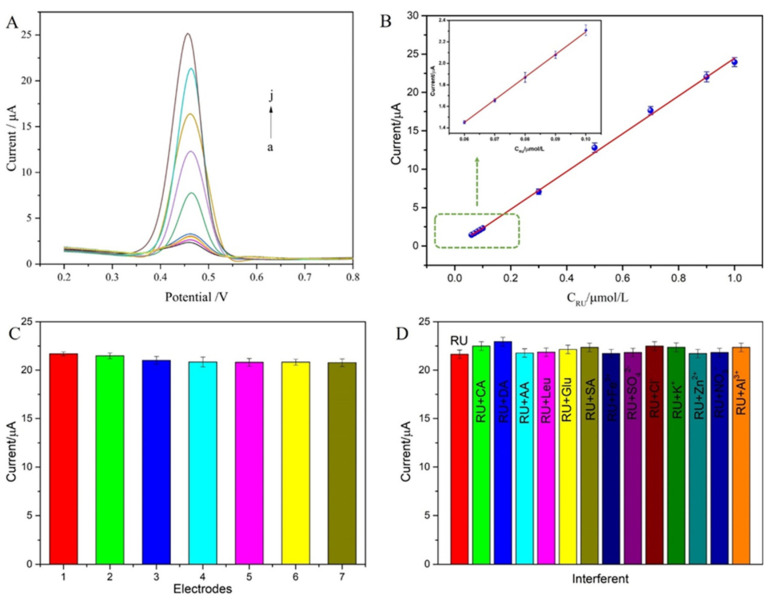
(**A**) DPVs obtained from β-CD-Ni-MOF-74/rGO/GCE in the presence of RU ranging from a to j (0.06, 0.07, 0.08, 0.09, 0.1, 0.3, 0.5, 0.7, 0.9, 1.0 μnmol/L) in 0.1 M PBS (pH 3.5); (**B**) The DPVs of linear calibration between the current responses and the concentration of RU. (**C**) Reproducibility study of seven individual electrodes in the same conditions. (**D**) Effect of some possible interfering substances on the determination of 2 μM RU in PBS solution.

Under the optimal detection conditions, the electrochemical response of β-CD-Ni-MOF-74/rGO-modified electrodes with different concentrations of RU (pH = 3.5) was studied using DPV technology. As shown in Figure 7A, as the RU concentration increased from 0.06 μM to 1.0 μM, The ipa gradually increased, and the LOD is 0.68 nM (S/N = 3). There are two linear relationships between RU concentration and ipa (Figure 7B) and the linear equations obtained:

ipa (μA) = 21.33 C (μM) + 0.1676 (0.06–0.1 µM, R^2^ = 0.9996)

ipa (μA) = 24.374 C (μM) + 0.0833 (0.10–1.0 µM, R^2^ = 0.9974)

Compared with the sensors shown in Table 1, the sensor we constructed has higher sensitivity and a lower detection limit. Therefore, the β-CD-Ni-MOF-74/rGO/GCE has sensitive detection for RU with a wider linear range. In addition, the materials we have prepared may have potential for other applications as electrochemical sensors.

### 2.7. Repeatability, Reproducibility, and Selectivity

In order to study the anti-interference ability of the sensor, various interfering substances were added to a 0.1 M BPS solution containing 1 µM RU (pH = 3.5). DPV technology was used to detect changes in peak current to verify the anti-interference ability of the sensor. As shown in Figure 7D, it can be seen that by adding 100 times the dosage of ions, Al^3+^, Mg^2+^, Fe^3+^, K^+^, Zn^2+^, SO_4_^2−^, NO_3_^−^, and Cl^−^ and 50 times the dosage of sucrose, glucose (Glu), L-leucine (Leu), salicylic acid (SA), ascorbic acid (AA), citric acid (CA), and dopamine hydrochloride (DA), the obtained ipa signal value changed by less than 10%. All the results indicate that β-CD/Ni-MOF-74/rGO/GCE has good selectivity for RU detection.

To evaluate the reproducibility of β-CD/Ni-MOF-74/rGO/GCE, seven independent electrodes were tested under the same conditions, and the relative standard deviation (RSD) was 5% (Figure 7C). We investigated the long-term stability of the modified electrode under the same conditions. Firstly, the ip of β-CD/Ni-MOF-74/rGO/GCE was obtained in 0.1 M PBS (1 µM RU, pH = 3.5), and then, after 30 days of storage, the ip of the above-modified electrode was obtained under the same conditions. The standard deviation of the above two current values was 0.6%, which indicates that the sensor has good long-term stability.

### 2.8. Detection of RU in Actual Sample

In this study, β-CD/Ni-MOF-74 was synthesized by using an in situ hydrothermal method, and a novel ultra-sensitive RU electrochemical sensor based on β-CD/Ni-MOF-74/rGO composite was proposed. Notably, having good conductivity, large specific surface area, and β-CD with good adsorption enrichment performance, the rGO/β-CD-Ni-MOF-74/GCE exhibited the high-performance detection of rutin. Due to the splendid electrochemical performance of rGO/β-CD-Ni-MOF-74, the sensor has been successfully applied to the detection of rutin tablets, showing satisfactory results and considerable application potential. This study presents an interesting technique for the use of β-CD-MOFs in electrochemical sensor studies, as well as a new method for the detection of RU.

Firstly, rutin tablets were added to a 0.1 M PBS solution (pH = 3.5). By using the standard addition method, different concentrations of RU standard solutions were sequentially added to the PBS solution (Table S1). The recovery rate was 93.9–100.7%, and the RSD was 0.11–0.21%. All the results indicated that the sensor we proposed is feasible and can be used for the detection of actual samples.

## 3. Experiment

### 3.1. Preparation of β-CD-Ni-MOF-74

Firstly, Ni(CH₃COO)₂·4H_2_O (0.747 g) and β-CD (0.747 g) were dissolved in 30 mL of deionized water and then 2,5-dihydroxyterephthalic acid (DHTA, 0.594 g) was dissolved in 30 mL tetrahydrofuran (THF). Next, the above two solutions were mixed and vigorously stirred for 10 min and then transferred to a high-pressure reactor at 110 °C for 24 h. After the reaction, the power was centrifuged, washed, and dried to obtain β-CD-Ni-MOF-74. MOF-74 was prepared by using the same method, except for the absence of β-CD [40].

### 3.2. Preparation of GO

Graphene oxide nanosheets (GO) were synthesized by modified Hummers and Offeman methods [51]. Firstly, graphite powder (6.0 g) and sodium nitrate (3.0 g) were mixed and slowly added into H_2_SO_4_. Then, 15.0 g KMnO_4_ was slowly added at 5 °C. After removing the ice bath device, the reaction was continued at room temperature and stirring was continued. Next, 200 mL of deionized water was added to the reaction solution, and the temperature was raised to 98 °C. Deionized water containing hydrogen peroxide was added to the reaction until the solution was light brown and left to sit overnight. After the reaction, the product was centrifuged at 9000 rpm for 10 min, washed with a large amount of DI water, and dried for 12 h at 60 °C in a vacuum. The final GO powder was then obtained.

### 3.3. Preparation of the Modified Electrodes

**Scheme 1:**
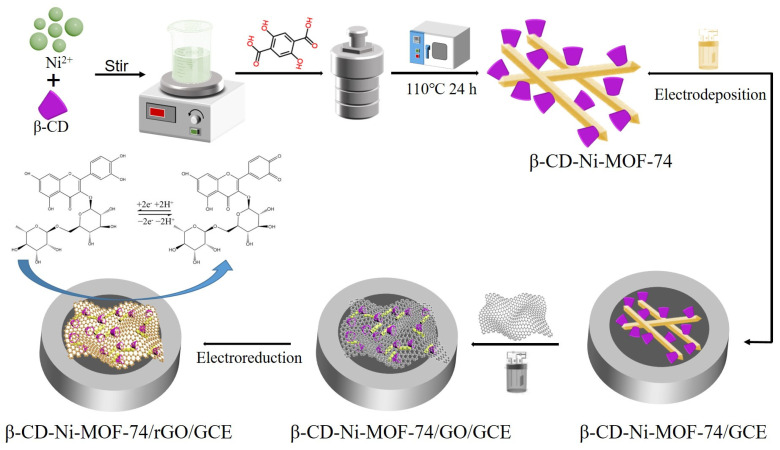
Schematic representation of the fabrication procedure of β-CD-Ni-MOF-74/rGO/GCE.

The glassy carbon electrode (GCE) was first polished with Al_2_O_3_ powder, followed by ultrasonic washing with ethanol and deionized water to remove impurities. Finally, the clean GCE was dried under an infrared light for use in the future. Different modified electrodes were prepared by electrodeposition and electrochemical reduction. Firstly, 2.0 mg of GO and β-CD-Ni-MOF-74 were ultrasonically dispersed in 10 mL PH buffer solution (PBS), respectively. Then, β-CD-Ni-MOF-74/GCE was obtained by an electrodeposition method (cyclic voltammetry: 25 cycles; voltage range: −1.0 to 1.0 V; sweep speed: 100 mV·s^−1^). Subsequently, β-CD-Ni-MOF-74/GO/GCE was obtained by an electrodeposition method (cyclic voltammetry: 10 cycles; voltage range: −1.0 to 1.0 V; sweep speed: 100 mV·s^−1^). Finally, the β-CD-Ni-MOF-74/rGO/GCE was obtained by electrochemical reduction in a 0.1 M PBS solution (voltage range: −0.9 to 0 V and sweep speed: 100 mV·s^−1^). For comparison, bare GCE, β-CD-Ni-MOF-74/GCE, β-CD/GCE, Ni-MOF-74/GCE, Ni-MOF-74/rGO/GCE, rGO/GCE, and β-CD/rGO/GCE were constructed by using the same method. A schematic representation of the fabrication procedure of β-CD-Ni-MOF-74/rGO/GCE is shown in Scheme 1.

### 3.4. Materials and Apparatus

Rutin was purchased from Shanghai Yuanye Biotechnology Co., Ltd., Shanghai, China. β-cyclodextrin and graphite powder were purchased from Shanghai Macklin Biochemical Co., Ltd., Shanghai, China. Manganese chloride (MnCl_2_·4H_2_O) was obtained from coriolis reagent. Nickel acetate (Ni(CH_3_COO)_2_·4H_2_O), 2,5-dihydroxyterephthalic acid (DHTA) were obtained from Aladdin Reagent Co., Ltd., Shanghai, China.

The electrochemical experiments were carried out in an electrolytic cell with a three-electrode system (CHI 660E electrochemical workstation, platinum wire counter electrode, Ag/AgCl reference electrode, and β-CD@MOF-74/rGO/GCE working electrode). The materials were characterized using a scanning electron microscope (SEM, Sigma HD ZEISS, Jena, Germany), X-ray photoelectron spectrum (XPS, SU8220 Hitachi, Tokyo, Japan), X-ray diffractometer (XRD, Bruker D8 Advanced, Billerica, MA, USA), and Fourier transform infrared spectrometer (FT-IR, Nicolet 6700, Waltham, MA, USA). In addition, the specific surface area and pore size distribution of the materials were calculated based on the data from a 3H-2000 nitrogen adsorption BET-specific surface tester of Beishide Instrument Co., Ltd. (Beijing, China).

### 3.5. Methods

Cyclic voltammetry (CV) curves were recorded at a scan rate of 50 mV/s on the working electrode within a range of −0.2 to 0.9 V. Electrochemical impedance spectroscopy (EIS) measurements were performed in a standard potassium ferricyanide solution system, of which the amplitude and pulse width were 50 mV and 5 mV, respectively,. Differential pulse voltammetry (DPV) was collected within 0.2–0.8 V for detecting RU. The electrode was electrochemically subjected to potential cycling between 0.2 and 0.9 V in phosphate buffer (pH 3.5) at 50 m V·s^−1^ until reaching a steady state before the next measurement. In EIS testing, the amplitude was 5 mV, with a frequency range of 0.01 Hz to 10^5^ Hz (the supporting electrolyte was a 5.0 mM [Fe(CN)_6_]^3−/4−^ solution containing 0.10 M KCl). Before all electrochemical tests, N_2_ must be applied in advance and the entire subsequent testing process must also be carried out in a nitrogen atmosphere.

### 3.6. Preparation of Actual Sample

Rutin tablets were purchased from the school hospital and used for the detection of rutin in real samples. An amount of 0.5 mg of rutin tablets was ground and dissolved in 10 mL of anhydrous ethanol using ultrasound.

## 4. Conclusions

In this work, β-CD-Ni-MOF-74 was synthesized by a hydrothermal method and compounded with GO by an electrochemical method to further synthesize β-CD-Ni-MOF-74/rGO composite materials. With the good conductivity of rGO and the large specific surface area and excellent enrichment ability of β-CD-Ni-MOF-74, the sensor achieved the sensitive detection of rutin in a wider linear range (0.06–1.0 μM) and at a lower detection limit (LOD, 0.68 nM, (S/N = 3)). In addition, the sensor achieved satisfactory results and recovery rates in the detection of actual samples.

## Figures and Tables

**Table 1:** Comparison of different modified electrodes for detecting RU.

Electrode	Linear Range (μM)	LOD (μM)	Technique	Ref.
Cu@C@CoO/GCE	0.01–10.0	0.418 × 10^−3^	DPV	[46]
GR/AuNPs/AN	0.08–0.1, 0.2–2.0	0.225 × 10^−3^	DPV	[47]
NS-rGO/AuNPs	0.0002–0.0014	0.067 × 10^−3^	DPV	[48]
MWNTsIL-Gel/GCE	0.072–0.006	0.02 × 10^−3^	DPV	[49]
GR-MnO_2_/CILE	0.01–500	2.73 × 10^−3^	DPV	[50]
β-CD/Ni-MOF-74/rGO/GCE	0.06–1.0	0.68 × 10^−3^	DPV	This Work

## Data Availability

Data are contained within the article.

## Supplementary Materials

The following supporting information can be downloaded at: https://www.mdpi.com/article/10.3390/molecules28124604/s1. Scheme S1. The reasonable electrochemical reaction mechanism of RU at β-CD-Ni-MOF-74/rGO/GCE. Figure S1. (A) CVs of β-CD-Ni-MOF-74/rGO/GCE at different pH (from a to k: 3.0, 3.5, 4.0, 4.5, 5.0, 5.5, 6.0, 6.5, 7.0, 7.5, 8.0) in 0.1 M PBS containing 2 μM RU. (B) The dependence of oxidation peak potential (Epa), formal potential (Eθ), reduction peak potential (Epc) on pH. (C) The linear relationship between redox current (ipa and ipc) and pH. Figure S2. (A) CVs of β-CD-Ni-MOF-74/rGO/GCE at different scan rates (from a to t: 20, 40, 60, 80, 100, 120, 140, 160, 180, 200, 220, 240, 260, 280, 300, 320, 340, 360, 380, 400 mV⋅s^−1^) in 0.1 M PBS (pH 3.5) containing 2 μM RU. (B) The dependence of redox peak currents on the scan rates (v). (C) The dependence of oxidation peak potential on lnv. Figure S3. (A) The DPV response of 1 μM RU on modified electrodes of different electrodeposition cycles. (B) Influence of accumulation potential on the oxidation peak current of 2 μM RU. (C) Influence of accumulation time on the oxidation peak current of 2 μM RU. Accumulation potential: 0.2 V. Table S1. Determination of RU in actual samples by β-CD-Ni-MOF-74/rGO/GCE sensor (n = 3).

## Sample Availability

Samples of the compounds are not available from the authors.
