# Supplementary material for: Electrochemical Behavior of β-Cyclodextrin-Ni-MOF-74/Reduced Graphene Oxide Sensors for the Ultrasensitive Detection of Rutin
Source: Molecules. 2023 Jun 7;28(12):4604. doi: 10.3390/molecules28124604 (PMC10303991; doi:10.3390/molecules28124604)
Supplement: Supplementary file 1 [file molecules-28-04604-s001.zip › molecules-2380603-supplementary.pdf]

Supplementary Material

Electrochemical Behavior of β-Cyclodextrin-Ni-MOF-74/Reduced Graphene Oxide Sensors for the Ultrasensitive Detection of Rutin

Li Zhang [1,2,3,4,†], Mengting Zhang [5,†], Pingping Yang [1], Yin Zhang [6], Junjie Fei [4,5,*] and Yixi Xie [2,3,5,*]

[1]Hunan Engineering Laboratory for Preparation Technology of Polyvinyl Alcohol Fiber Material, College of Chemistry and Materials Engineering, Huaihua University, Huaihua 418000, China

[2]Key Laboratory of Research and Utilization of Ethnomedicinal Plant Resources of Hunan Province, Huaihua University, Huaihua 418008, China

[3]Hunan Provincial Higher Education Key Laboratory of Intensive Processing Research on Mountain Ecological Food, Huaihua 418008, China

[4]Key Laboratory of Environmentally Friendly Chemistry and Applications of Ministry of Education, College of Chemistry, Xiangtan University, Xiangtan 411105, China

[5]Key Laboratory for Green Organic Synthesis and Application of Hunan Province, Xiangtan University, Xiangtan 411105, China

[6]Junior Education Department, Changsha Normal University, Changsha 410100, China

*Correspondence: fei_junjie@xtu.edu.cn (J.F.); xieyixige@xtu.edu.cn (Y.X.);

Tel.: +86-731-5829-2060 (J.F.)

†These authors contributed equally to this work.

List of Supplementary Tables and Figures:

Scheme S1. The reasonable electrochemical reaction mechanism of RU at β-CD-Ni-MOF-74/rGO/GCE.

Figure S1. (A) CVs of β-CD-Ni-MOF-74/rGO/GCE at different pH (from a to k: 3.0, 3.5, 4.0, 4.5, 5.0, 5.5, 6.0, 6.5, 7.0, 7.5, 8.0) in 0.1 M PBS containing 2 μM RU. (B) The dependence of oxidation peak potential (Epa), formal potential (E^{θ}), reduction peak potential (Epc) on pH. (C) The linear relationship between redox current (ipa and ipc) and pH.

Figure 2. (A) CVs of β-CD-Ni-MOF-74/rGO/GCE at different scan rates (from a to t: 20, 40, 60, 80, 100, 120, 140, 160, 180, 200, 220, 240, 260, 280, 300, 320, 340, 360, 380, 400 $mV \cdot s^{-1}$) in 0.1 M PBS (pH 3.5) containing 2 μM RU. (B) The dependence of redox peak currents on the scan rates (*v*). (C) The dependence of oxidation peak potential on ln*v*.

Figure S3. (A) The DPV response of 1 μM RU on modified electrodes of different electrodeposition cycles. (B) Influence of accumulation potential on the oxidation peak current of 2 μM RU. (C) Influence of accumulation time on the oxidation peak current of 2 μM RU. Accumulation potential: 0.2 V.

Table S1. Determination of RU in actual samples by β-CD-Ni-MOF-74/rGO/GCE sensor (n=3).

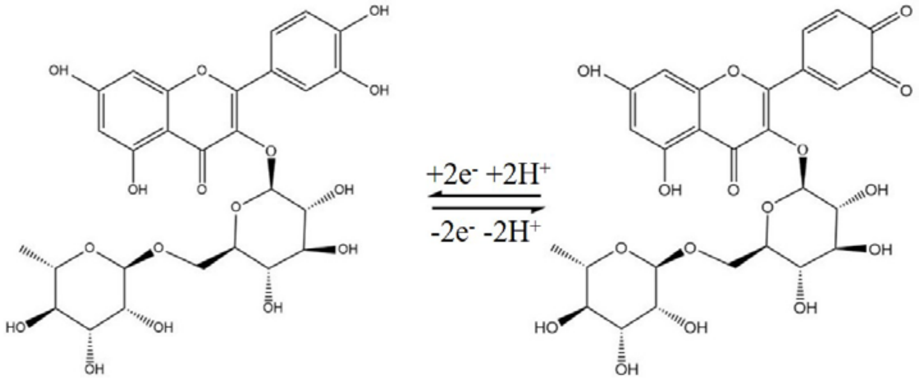

Scheme S1

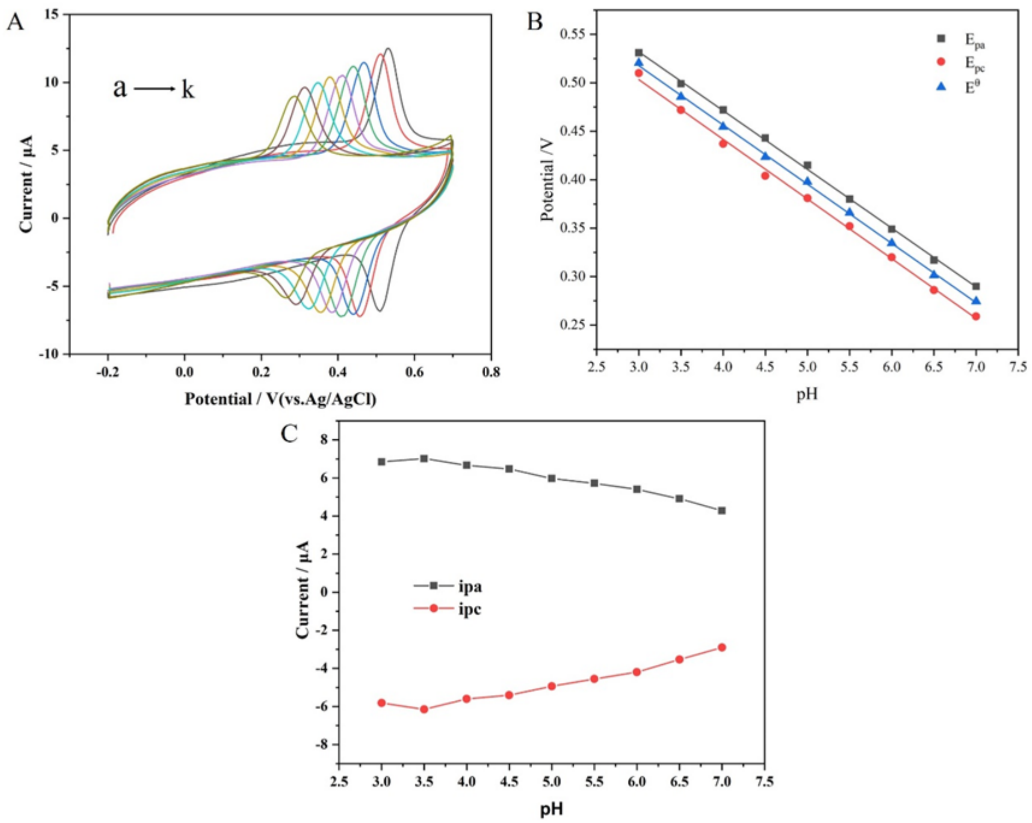

Figure S1

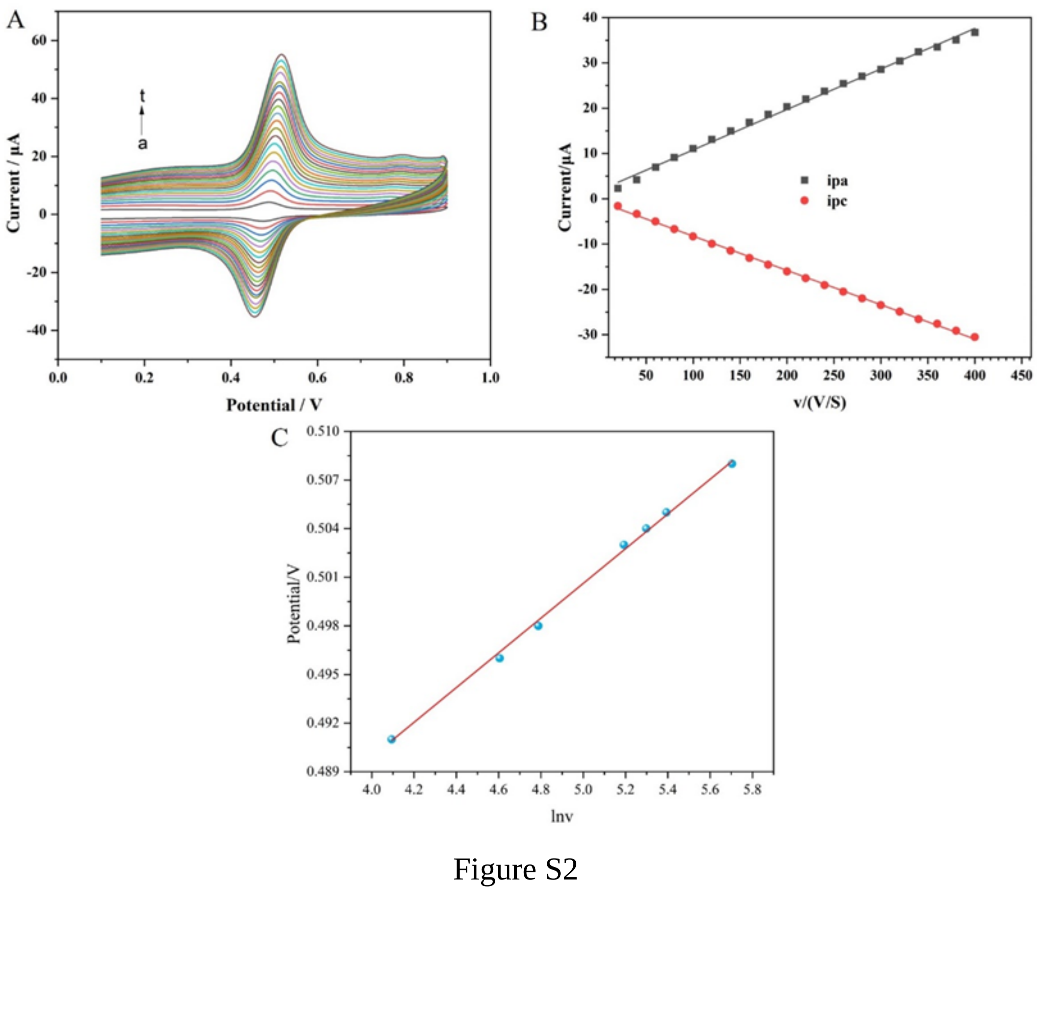

Figure S2

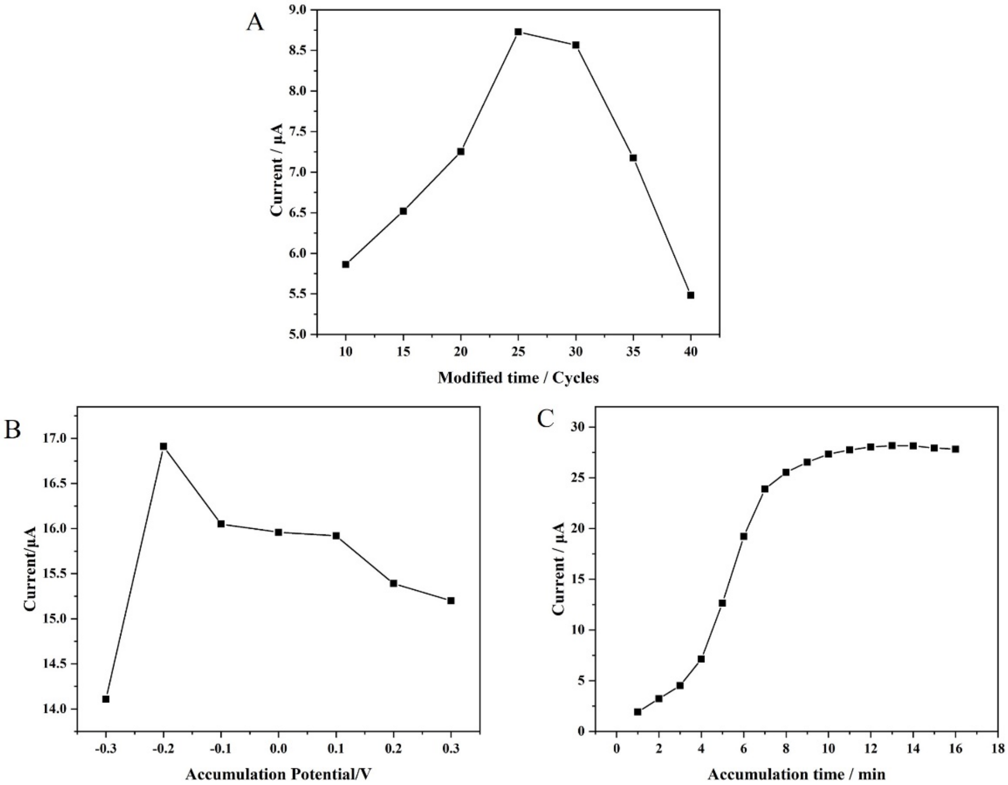

Figure S3

Table S1

	Added (μM)	Founded (μM)	Recovery (%)	RSD (%)
	0	0.055	—	—
rutin	0.05	0.1033	96.4	0.21
tablet	0.05	0.1556	101	0.11
	0.05	0.2075	104.5	0.16
